# Supplementary material for: AI Naturalists Might Hold the Key to Unlocking Biodiversity Data in Social Media Imagery
Source: Patterns (N Y). 2020 Oct 9;1(7):100116. doi: 10.1016/j.patter.2020.100116 (PMC7660428; doi:10.1016/j.patter.2020.100116)
Supplement: Document S1. Supplemental Experimental Procedures [file mmc1.pdf]

PATTER, Volume *1*

Supplemental Information

AI Naturalists Might Hold the Key to Unlocking Biodiversity Data in Social Media Imagery

Tom A. August, Oliver L. Pescott, Alexis Joly, and Pierre Bonnet

Accessing and Identifying Images

Introduction

In this document we outline the methods used to collate images from Flickr, and analyse images using Pl@ntNet, used in the paper “August et al. 2020. AI naturalists might hold the key to unlocking biodiversity data in social media imagery. Patterns”.

This code heavily relies on two R packages, `photosearcher` and `plantnet`. Here we show the methods used in the paper as well as some simpler cases to allow demonstration. It is important to note that with the ever changing data on Flickr and the ever changing model behind the Pl@ntNet API, it will not be possible to exactly replicate our analysis even if using the same code.

Accessing images

Images were accessed using the `photosearcher` package. At the time of writing this is available at: https://github.com/ropensci/photosearcher, and is accompanied by tutorials and help documentation. We performed two searches with this function, one for each of the target survey areas. These searches can take some time to run and require the user to first set up their user tokens, described in the package help documentation.

```
# Get the image data for London
photo_meta1 <- photo_search(mindate_taken = "2000-03-01",
                            maxdate_taken = "2019-10-01",
                            text = "flower",
                            bbox = "-2.021484,53.019740,-1.533966,53.603914")

# Get the image data for the Peak District
photo_meta2 <- photo_search(mindate_taken = "2000-03-01",
                            maxdate_taken = "2019-10-01",
                            text = "flower",
                            bbox = "-0.312836,51.439050,-0.005219,51.590237")
```

For demonstration purposes here is a small example for the London data on a single day

```
# Install the package from GitHub
devtools::install_github("ropensci/photosearcher")

## Skipping install of 'photosearcher' from a github remote, the SHA1
(65a9f488) has not changed since last install.
##   Use `force = TRUE` to force installation

library(photosearcher)

# Get the image data for London
demo <- photo_search(mindate_taken = "2019-10-01",
```

```
                     maxdate_taken = "2019-10-30",
                     text = "flower",
                     bbox = "-2.021484,53.019740,-1.533966,53.603914")
str(demo)
```

```
## 'data.frame':    5 obs. of  62 variables:
##  $ license              : int  0 0 0 0 1
##  $ id                   : num  4.89e+10 4.88e+10 4.88e+10 4.89e+10 4.89e+10
##  $ owner                : chr  "16819123@N06" "16819123@N06" "16819123@N06"
"16819123@N06" ...
##  $ secret               : chr  "578f9e80f7" "457b740e9d" "d16cbec8b0"
"2971847ee0" ...
##  $ server               : num  65535 65535 65535 65535 65535
##  $ farm                 : num  66 66 66 66 66
##  $ title                : chr  "OrangeGlow" "Sunlit" "Wall" "Contemplation"
...
##  $ ispublic             : logi  NA NA NA NA NA
##  $ isfriend             : logi  NA NA NA NA NA
##  $ isfamily             : logi  NA NA NA NA NA
##  $ dateupload           : POSIXct, format: "2019-10-13 11:49:38" "2019-10-
03 11:56:09" ...
##  $ lastupdate           : POSIXct, format: "2020-04-06 23:51:03" "2019-11-
03 17:16:17" ...
##  $ datetaken            : POSIXct, format: "2019-10-02 13:49:21" "2019-10-
02 13:52:23" ...
##  $ datetakengranularity: int  0 0 0 0 0
##  $ datetakenunknown    : int  0 0 0 0 0
##  $ count_views          : int  759 740 764 1280 168
##  $ count_faves          : int  23 14 17 8 1
##  $ count_comments       : int  3 1 0 0 0
##  $ tags                 : chr  "nikon d7100 sigma 70mm orange orton
ortoneffect flowers surreal meerbrook staffs staffordshire abstract" "niko
d7100 sigma 70mm flower orange chiaroscuro churchyard grindon staffs
staffordshire" "nikon d7100 nikkor 55300mm wall whitewash flowers
hangingbasket window grindon staffs staffordshire" "nikon d600 nikkor 50mm
f18g flowers lily lilies whiteflowers stilllife church longnor staffs
staffordshire" ...
##  $ latitude             : num  53.1 53.1 53.1 53.2 53.2
##  $ longitude            : num  -2.02 -1.87 -1.87 -1.87 -1.63
##  $ accuracy             : int  16 16 15 16 16
##  $ context              : int  0 0 0 0 0
##  $ place_id             : chr  "" "" "" "" ...
##  $ woeid                : num  2642828 2647866 2647866 2643650 2650241
##  $ geo_is_public        : logi  NA NA NA NA NA
##  $ geo_is_contact       : logi  NA NA NA NA NA
##  $ geo_is_friend        : logi  NA NA NA NA NA
##  $ geo_is_family        : logi  NA NA NA NA NA
##  $ url_sq               : chr
"https://live.staticflickr.com/65535/48890416561_578f9e80f7_s.jpg"
"https://live.staticflickr.com/65535/48836184833_457b740e9d_s.jpg"
```

```
"https://live.staticflickr.com/65535/48836214616_d16cbec8b0_s.jpg"
"https://live.staticflickr.com/65535/48929255968_2971847ee0_s.jpg" ...
##  $ height_sq           : num  75 75 75 75 75
##  $ width_sq            : num  75 75 75 75 75
##  $ url_t               : chr
"https://live.staticflickr.com/65535/48890416561_578f9e80f7_t.jpg"
"https://live.staticflickr.com/65535/48836184833_457b740e9d_t.jpg"
"https://live.staticflickr.com/65535/48836214616_d16cbec8b0_t.jpg"
"https://live.staticflickr.com/65535/48929255968_2971847ee0_t.jpg" ...
##  $ height_t            : num  67 67 67 67 67
##  $ width_t             : num  100 100 100 100 100
##  $ url_s               : chr
"https://live.staticflickr.com/65535/48890416561_578f9e80f7_m.jpg"
"https://live.staticflickr.com/65535/48836184833_457b740e9d_m.jpg"
"https://live.staticflickr.com/65535/48836214616_d16cbec8b0_m.jpg"
"https://live.staticflickr.com/65535/48929255968_2971847ee0_m.jpg" ...
##  $ height_s            : num  160 160 160 160 160
##  $ width_s             : num  240 240 240 240 240
##  $ url_q               : chr
"https://live.staticflickr.com/65535/48890416561_578f9e80f7_q.jpg"
"https://live.staticflickr.com/65535/48836184833_457b740e9d_q.jpg"
"https://live.staticflickr.com/65535/48836214616_d16cbec8b0_q.jpg"
"https://live.staticflickr.com/65535/48929255968_2971847ee0_q.jpg" ...
##  $ height_q            : num  150 150 150 150 150
##  $ width_q             : num  150 150 150 150 150
##  $ url_m               : chr
"https://live.staticflickr.com/65535/48890416561_578f9e80f7.jpg"
"https://live.staticflickr.com/65535/48836184833_457b740e9d.jpg"
"https://live.staticflickr.com/65535/48836214616_d16cbec8b0.jpg"
"https://live.staticflickr.com/65535/48929255968_2971847ee0.jpg" ...
##  $ height_m            : num  333 333 333 334 333
##  $ width_m             : num  500 500 500 500 500
##  $ url_n               : chr
"https://live.staticflickr.com/65535/48890416561_578f9e80f7_n.jpg"
"https://live.staticflickr.com/65535/48836184833_457b740e9d_n.jpg"
"https://live.staticflickr.com/65535/48836214616_d16cbec8b0_n.jpg"
"https://live.staticflickr.com/65535/48929255968_2971847ee0_n.jpg" ...
##  $ height_n            : num  213 213 213 214 213
##  $ width_n             : num  320 320 320 320 320
##  $ url_z               : chr
"https://live.staticflickr.com/65535/48890416561_578f9e80f7_z.jpg"
"https://live.staticflickr.com/65535/48836184833_457b740e9d_z.jpg"
"https://live.staticflickr.com/65535/48836214616_d16cbec8b0_z.jpg"
"https://live.staticflickr.com/65535/48929255968_2971847ee0_z.jpg" ...
##  $ height_z            : num  427 427 427 427 427
##  $ width_z             : num  640 640 640 640 640
##  $ url_c               : chr
"https://live.staticflickr.com/65535/48890416561_578f9e80f7_c.jpg"
"https://live.staticflickr.com/65535/48836184833_457b740e9d_c.jpg"
"https://live.staticflickr.com/65535/48836214616_d16cbec8b0_c.jpg"
```

```
"https://live.staticflickr.com/65535/48929255968_2971847ee0_c.jpg" ...
##  $ height_c            : num  533 533 533 534 533
##  $ width_c             : num  800 800 800 800 800
##  $ url_l               : chr
"https://live.staticflickr.com/65535/48890416561_578f9e80f7_b.jpg"
"https://live.staticflickr.com/65535/48836184833_457b740e9d_b.jpg"
"https://live.staticflickr.com/65535/48836214616_d16cbec8b0_b.jpg"
"https://live.staticflickr.com/65535/48929255968_2971847ee0_b.jpg" ...
##  $ height_l            : num  683 683 683 684 683
##  $ width_l             : num  1024 1024 1024 1024 1024
##  $ url_o               : chr  NA NA NA NA ...
##  $ height_o            : num  NA NA NA NA 3648
##  $ width_o             : num  NA NA NA NA 5472
##  $ description         : chr  "An Orton effect image, achieved by
subtracting 30% of the original from a very blurred version.\nIn the
churchy"| __truncated__ "Flowers starkly picked out by a shaft of sunlight in
the churchyard at Grindon, Staffordshire.\nCamera set at -"| __truncated__
"The wall of house in Grindon, Staffordshire" "Lilies in the church at
Longnor, Staffordshire" ...
##  $ license_name        : chr  "All Rights Reserved" "All Rights Reserved"
"All Rights Reserved" "All Rights Reserved" ...
##  $ license_url         : chr  "NA" "NA" "NA" "NA" ...
```

As you can see a large amount of metadata is returned including links to the images at various sizes.

Image identification

Once you have the URLs for the images of interest these can be passed to the `plantnet` package for classification. The `plantnet` package is available on GitHub and has tutorials and help documentation - https://github.com/BiologicalRecordsCentre/plantnet

To use this API you need to create a key on the Pl@ntNet API pages - https://my.plantnet.org/

```
# Install the package from GitHub
devtools::install_github("BiologicalRecordsCentre/plantnet")

## Skipping install of 'plantnet' from a github remote, the SHA1 (260601c7)
has not changed since last install.
##   Use `force = TRUE` to force installation

library(plantnet)

##
## Attaching package: 'plantnet'

## The following object is masked from 'package:graphics':
##
##     identify
```

```r
# Lets take a look at one of the images
download.file(demo$url_l[4],
              'temp.jpg', mode = 'wb')
jj <- readJPEG('temp.jpg', native = TRUE)
plot(0:1, 0:1, type = "n", ann = FALSE, axes = FALSE)
rasterImage(jj,0,0,1,1)
```

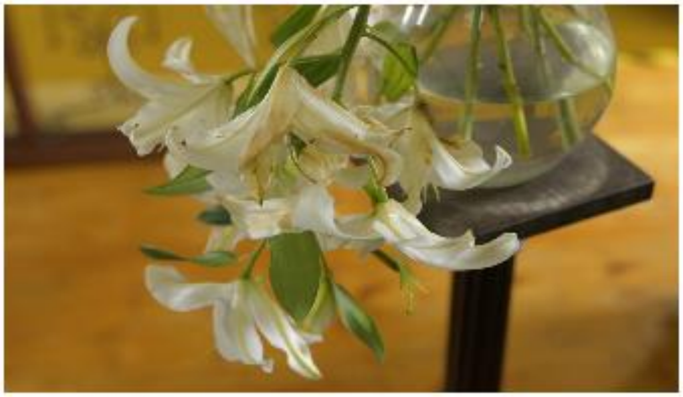

```r
# Now lets identify this image
id <- identify(key = Secret_Key,
               imageURL = demo$url_l[4])
id

##        score     latin_name                common_name
##  [1,] 15.67988  "Lilium candidum"         "Madonna lily"
##  [2,] 3.226063  "Lilium longiflorum"      "Easter lily"
##  [3,] 2.911383  "Allium triquetrum"       "Triquetrous garlic"
##  [4,] 2.354943  "Robinia pseudoacacia"    "False acacia"
##  [5,] 2.086884  "Ornithogalum nutans"     "Drooping star-of-Bethlehem"
##  [6,] 1.824277  "Galega officinalis"      "Goat's-rue"
##  [7,] 1.781997  "Dendrobium nobile"       "Dendrobium Stem"
##  [8,] 1.01721   "Uvularia grandiflora"    "Bellwort"
##  [9,] 0.8832858 "Hosta plantaginea"       "August-lily"
## [10,] 0.7705645 "Polygonatum odoratum"    "Solomon's-seal"
## [11,] 0.65286   "Hyacinthus orientalis"   "Hyacinth"
## [12,] 0.5609712 "Moringa oleifera"        "Drumsticktree"
## [13,] 0.5388624 "Pancratium maritimum"    "Sea-daffodil"
```

```
## [14,] 0.5148856 "Eucharis × grandiflora" NA
## [15,] 0.500501  "Paradisea liliastrum"   "St. Bruno's Lily"
```

Details on what is returned is given in the package help. In brief the score is an indication of the confidence the classifier has in its own identification. The top species is the species the classifier is most confident is represented in the image. In our study we only considered the top identification given to each image.

Image review

In our paper we undertake expert assessment of the images and the classifications in order to critically assess biases in both the images available on Flickr and the biases in the image classifier. We do this by creating a Shiny application that allowed a trained botanist to quickly go through the images and create a dataset of information about the image and the accuracy of the classification. Below is a screen shot of the app and the code used to create it.

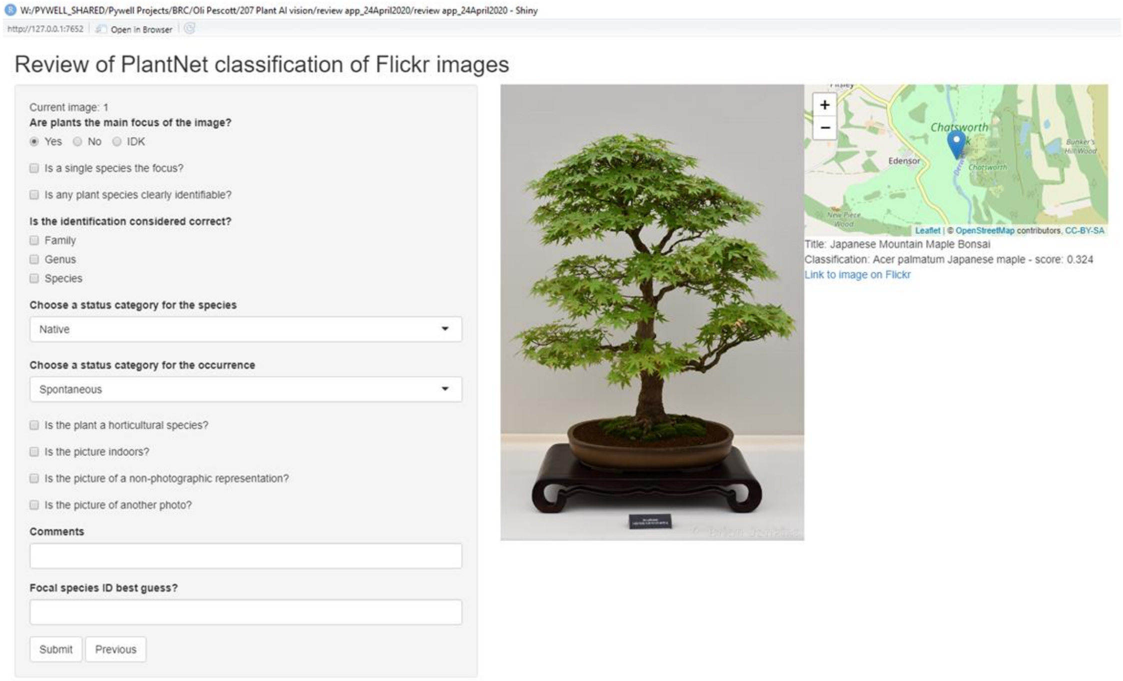

Screenshot of shiny app

```
# # Add a random number column to sample images from
# # This is added the the raw data returned from Flickr
# ids <- read.csv('data/id_results_peaks_cleaned.csv',
#                 stringsAsFactors = FALSE,
#                 header = TRUE)
# ids$rand_id <- sample(1:nrow(ids),
#                       size = nrow(ids),
#                       replace = FALSE)
# write.csv(ids,
#           file = 'data/id_results_peaks_cleaned.csv',
```

```
#           row.names = FALSE)

# Load in the data returned from Flickr
ids <- read.csv('data/id_results_peaks_cleaned.csv',
                stringsAsFactors = FALSE,
                header = TRUE)
ids <- dplyr::distinct(ids)

# Load in the table we are writing results to
# Formatted as in the excel supplementary materials
# to August et al 2020.
results <- 'results/review_results.csv'

library(shiny)
library(leaflet)

ui <- fluidPage(

    # Application title
    titlePanel("Review of PlantNet classification of Flickr images"),

    sidebarLayout(
        sidebarPanel(
            textOutput('imgID'),
            radioButtons(inputId = 'plantFocus',
                         inline = TRUE,
                         label = 'Are plants the main focus of the image?',
                         choices = c('Yes', 'No', 'IDK')),
            checkboxInput(inputId = 'plantFocSingle',
                          label = 'Is a single species the focus?',
                          value = FALSE),
            checkboxInput(inputId = 'plantIDable',
                          label = 'Is any plant species clearly
identifiable?',
                          value = FALSE),
            checkboxGroupInput(inputId = 'plantIDcorrect',
                               label = 'Is the identification considered
correct?',
                               choices = c('Family', 'Genus', 'Species')),
            selectInput(inputId = 'status_spp',
                        label = 'Choose a status category for the species',
                        choices = c('Native', 'Archaeophyte', 'Neophyte',
'NA')),
            selectInput(inputId = 'status_occ',
                        label = 'Choose a status category for the
occurrence',
                        choices = c('Spontaneous', 'Introduced', 'Unknown',
'NA')),
            checkboxInput(inputId = 'plantHort',
```

```
                          label = 'Is the plant a horticultural species?',
                          value = FALSE),
            checkboxInput(inputId = 'plantIndoor',
                          label = 'Is the picture indoors?',
                          value = FALSE),
            checkboxInput(inputId = 'plantRep',
                          label = 'Is the picture of a non-photographic
representation?',
                          value = FALSE),
            checkboxInput(inputId = 'plantMeta',
                          label = 'Is the picture of another photo?',
                          value = FALSE),
            textInput(inputId = 'comment',
                      label = 'Comments'),
            textInput(inputId = 'expertSpp',
                      label = 'Focal species ID best guess?'),
            actionButton(inputId = 'submit',
                         label = 'Submit'),
            actionButton(inputId = 'previous',
                         label = 'Previous')

        ),

        mainPanel(
           div(uiOutput("speciesImage"), style = 'float: left;'),
           leafletOutput('map', height = '200px', width = '400px'),
           textOutput('title'),
           textOutput('name'),
           htmlOutput('link')

        )
    )
)

server <- function(input, output, session) {

    imgID <- reactiveVal()
    if(file.exists(results)){
      so_far <- read.csv(results,
                         header = TRUE,
                         stringsAsFactors = FALSE)
      maxID <- min(ids$rand_id[ids$rand_id > max(as.numeric(so_far$rand_id),
                                                  na.rm = TRUE)])
      imgID(maxID)
    } else {
      imgID(min(ids$rand_id))
    }

    output$imgID <- renderText({
```

```
        paste('Current image:', imgID())
    })

    observeEvent(input$previous, {

      imgID(max(ids$rand_id[ids$rand_id < imgID()]))

    })

    # Output image title
    output$title <- renderText({

      paste('Title:', ids$title[ids$rand_id == imgID()])

    })

    # Output the displayed image name and score
    output$name <- renderText({

      x <- ids[ids$rand_id == imgID(),
               c('latin_name',
                 'common_name_english',
                 'classification_score')]
      paste('Classification:', x[1,1],
            x[1,2], '- score:',
            round(x[1,3],3))
    })

    # Output link to image
    output$link <- renderUI({
      a(href = as.character(ids$image_information_link[ids$rand_id ==
imgID()]),
        'Link to image on Flickr')
    })

    # Mini-map of location
    output$map <- renderLeaflet({

      x <- ids[ids$rand_id == imgID(), ]

      if(nrow(x) > 0){

        leaflet() %>%
          addTiles() %>%
          addMarkers(lng = x$longitude,
                     lat = x$latitude)

      }
    })
```

```r
observeEvent(input$submit, {

  done <- read.csv(results)

  if(!imgID() %in% done$rand_id){

    x <- ids[ids$rand_id == imgID(), ]
    x$plantFocus <- input$plantFocus
    x$plantFocSingle <- input$plantFocSingle
    x$plantIDable <- input$plantIDable
    x$plantIDcorrect <- paste(input$plantIDcorrect, collapse = ';')
    x$status_spp <- input$status_spp
    x$status_occ <- input$status_occ
    x$plantHort <- input$plantHort
    x$plantIndoor <- input$plantIndoor
    x$plantRep <- input$plantRep
    x$plantMeta <- input$plantMeta
    x$comment <- input$comment
    x$expertSpp <- input$expertSpp

    write.table(x,
                file = results,
                append = TRUE,
                col.names = FALSE,
                row.names = FALSE,
                sep = ',')

    nv <- min(ids$rand_id[ids$rand_id > imgID()])
    imgID(nv)

  } else {

    done[done$rand_id == imgID(),
         c('plantFocus', 'plantFocSingle','plantIDable',
           'plantIDcorrect','status_spp',
           'status_occ', 'plantHort','plantIndoor',
           'plantRep', 'plantMeta',
           'comment', 'expertSpp')] <- c(input$plantFocus,
                                         input$plantFocSingle,
                                  input$plantIDable,
                                  input$plantIDcorrect,
                                  input$status_spp,
                                  input$status_occ,
                                  input$plantHort,
                                  input$plantIndoor,
                                  input$plantRep,
                                  input$plantMeta,
                                  input$comment,
```

```
                                input$expertSpp)

        write.table(done,
                    file = results,
                    append = FALSE,
                    col.names = TRUE,
                    row.names = FALSE,
                    sep = ',')

        nv <- min(ids$rand_id[ids$rand_id > imgID()])
        imgID(nv)
      }

      updateRadioButtons(session = session,
                         inputId = 'plantFocus',
                         inline = TRUE,
                         label = 'Are plants the main focus of the image?',
                         choices = c('Yes', 'No', 'IDK'))
      updateCheckboxInput(session = session,
                          inputId = 'plantFocSingle',
                          label = 'Is a single species the focus?',
                          value = FALSE)
      updateCheckboxGroupInput(session = session,
                               inputId = 'plantIDcorrect',
                               choices = c('Family', 'Genus', 'Species'))
      updateCheckboxInput(session = session,
                          inputId = 'plantIDable',
                          label = 'Is a plant species identifiable?',
                          value = FALSE)
      updateCheckboxInput(session = session,
                          inputId = 'plantHort',
                          label = 'Is the plant a horticultural species?',
                          value = FALSE)
      updateCheckboxInput(session = session,
                          inputId = 'plantIndoor',
                          label = 'Is the picture indoors?',
                          value = FALSE)
      updateCheckboxInput(session = session,
                          inputId = 'plantRep',
                          label = 'Is the picture of a non-photographic
representation?',
                          value = FALSE)
      updateCheckboxInput(session = session,
                          inputId = 'plantMeta',
                          label = 'Is the picture of another photo?',
                          value = FALSE)
      updateTextInput(session = session,
                      inputId = 'comment',
                      label = 'Comments',
                      value = '')
```

```
      updateTextInput(session = session,
                      inputId = 'expertSpp',
                      label = 'Focal species ID best guess?',
                      value = '')
      updateSelectInput(session = session,
                        inputId = 'status_spp',
                        label = 'Choose a status category for the species',
                        choices = c('Native', 'Archaeophyte', 'Neophyte',
'NA'))
      updateSelectInput(session = session,
                        inputId = 'status_occ',
                        label = 'Choose a status category for the
occurrence',
                        choices = c('Spontaneous', 'Introduced', 'Unknown',
'NA'))

    })

    output$speciesImage <- renderUI({
        div(id = 'spImageDiv',
            img(src = as.character(ids$url_large_image[ids$rand_id ==
imgID()]),
                style = 'max-width: 600px; max-height: 600px')
        )
    })
}

# Run the application
shinyApp(ui = ui, server = server)
```
