# Supplementary material for: AI Naturalists Might Hold the Key to Unlocking Biodiversity Data in Social Media Imagery
Source: Patterns (N Y). 2020 Oct 9;1(7):100116. doi: 10.1016/j.patter.2020.100116 (PMC7660428; doi:10.1016/j.patter.2020.100116)
Supplement: Document S2. Article plus Supplemental Information [file mmc3.pdf]

Article

Patterns

AI Naturalists Might Hold the Key to Unlocking Biodiversity Data in Social Media Imagery

Highlights

- AI image classifiers can create biodiversity datasets from social media imagery
- Flickr hosts many images of plants; some can be accurately classified to species by AI
- Images are spatially aggregated around tourist sites and under-represent native species
- Images focused on a single, non-horticultural, plant are most reliably identified

Authors

Tom A. August, Oliver L. Pescott, Alexis Joly, Pierre Bonnet

Correspondence

tomaug@ceh.ac.uk

In Brief

We apply newly developed AI image classifiers to large social media image datasets in order to assess whether new datasets of biodiversity observations can be generated in this way. We explore biases in both the dataset of images as well as in the ability of the AI image classifier to make accurate identifications and propose a checklist of questions researchers should ask themselves when considering this approach to data generation.

August et al., 2020, Patterns *1*, 100116
October 9, 2020 © 2020 The Authors.
https://doi.org/10.1016/j.patter.2020.100116

CellPress

Patterns

CellPress
OPEN ACCESS

Article

AI Naturalists Might Hold the Key to Unlocking Biodiversity Data in Social Media Imagery

Tom A. August,[1,5,*] Oliver L. Pescott,[1] Alexis Joly,[2] and Pierre Bonnet[3,4]
[1]UK Centre for Ecology and Hydrology, Benson Lane, Crowmarsh Gifford, Wallingford, Oxfordshire OX10 8BB, UK
[2]INRIA Sophia-Antipolis - ZENITH Team, LIRMM - UMR 5506 - CC 477, 161 Rue Ada, 34095 Montpellier Cedex 5, France
[3]AMAP, Univ Montpellier, CIRAD, CNRS, INRA, IRD, Montpellier, France
[4]CIRAD, UMR AMAP, Montpellier, France
[5]Lead Contact
*Correspondence: tomaug@ceh.ac.uk
https://doi.org/10.1016/j.patter.2020.100116

THE BIGGER PICTURE Recent reports of global biodiversity decline make it more important than ever to monitor biodiversity so that we can detect changes and infer their drivers. Online digital media, such as social media images, may be a new source of biodiversity observations, but they are far too numerous for a human to practically review. In this paper we apply an AI image classifier, designed to identify plants from images, to social media imagery to assess this method as a way to generate new biodiversity observations. We find that this approach is able to generate new data on species occurrence but that there are biases in both the social media data and the AI image classifier that need to be considered in analyses. This approach could be applied outside the biodiversity domain, to any phenomena of interest that may be captured in social media imagery. The checklist we provide at the end of this paper should therefore be of interest to anyone considering this approach to generating new data.

Proof-of-Concept: Data science output has been formulated, implemented, and tested for one domain/problem

SUMMARY

The increasing availability of digital images, coupled with sophisticated artificial intelligence (AI) techniques for image classification, presents an exciting opportunity for biodiversity researchers to create new datasets of species observations. We investigated whether an AI plant species classifier could extract previously unexploited biodiversity data from social media photos (Flickr). We found over 60,000 geolocated images tagged with the keyword "flower" across an urban and rural location in the UK and classified these using AI, reviewing these identifications and assessing the representativeness of images. Images were predominantly biodiversity focused, showing single species. Non-native garden plants dominated, particularly in the urban setting. The AI classifier performed best when photos were focused on single native species in wild situations but also performed well at higher taxonomic levels (genus and family), even when images substantially deviated from this. We present a checklist of questions that should be considered when undertaking a similar analysis.

INTRODUCTION

The ever-growing number of digital sensors in the environment has led to an increase in the amount of digital data being generated. This includes data from satellites, weather stations, data from "internet of things" devices, and data collected by members of the public via smartphone applications, to name but a few. These new sources of data have contributed to the era of "Big Data" characterized by large volumes of data, of numerous types and quality, being generated at an increasing speed.[1] This presents challenges and opportunities across a number of domains, including water management,[2] camera trapping,[3] and acoustic[4] analysis. To process these data into useful information there are many tools available, including classical statistical analyses[5] and classification by citizen scientists.[6] However, at some point traditional approaches may become inefficient or even impossible given the volume, diversity, and heterogeneity of these data. Storage, exploration, curation, and revision of data may have to be re-thought to allow for their quick and efficient transformation, annotation, or analysis. This is particularly

This is an open access article under the CC BY license (http://creativecommons.org/licenses/by/4.0/).

difficult for multimedia data which are typically much more complex than other data types. For example, biodiversity and environmental records in the form of audio, video, or image files are typically larger and more complex than text or numeric data. Large-scale analysis of multimedia data has only been possible in recent years since the development of large computational facilities, both academic and commercial. Regardless, the analysis of multimedia data is often further complicated because of their non-standardized methods of acquisition, with highly diverse devices, sensors, formats, scales, environmental contexts, and taxonomic scope. Building efficient, scalable, and robust approaches to solve these problems is a difficult scientific challenge at the forefront of data science and machine learning specifically.

Artificial intelligence (AI) techniques have profoundly transformed our ability to extract information from visual data. AI techniques have been applied for a long time in security and industrial domains, for example, in iris recognition[7] or the detection of faulty objects in manufacturing.[8] They were nevertheless only recently made more widely accessible after their use in smartphone apps for face recognition[9] and song identification.[10] Combined with increasing access to cloud-based computation, AI techniques can now automatically analyze hundreds of thousands of visual data every day.

AI can also be used to extract information from big data in order to address various challenges faced by society. For example, in conservation biology there is a pressing need to understand the state of our natural environment, and the drivers of observed declines in biodiversity.[11] In addition, signatories to the Convention on Biological Diversity have an obligation to monitor their biodiversity under Article 7.[12] In a number of nations, the monitoring of biodiversity is supported by long-running citizen science activities;[13] indeed, contributions from amateur naturalists to biodiversity data collection date back at least to the 19th century[14,15] (before this the distinction between amateur and professional scientists is blurred). However, recent years have arguably represented a significant shift in the amount of data collected by volunteer observers,[16] and in many cases observations are now accompanied by a digital image of the observation. These images are often verified by other observers (e.g., iNaturalist, www.inaturalist.org) or by a designated group of experts (e.g., iRecord, www.brc.ac.uk/irecord). Citizen-collected image data have clearly contributed considerable amounts of data[17] to the global biodiversity monitoring effort, but we also note that images are not necessarily required for robust amateur contributions in this area. For example, the British and Northern Irish taxon-focused organizations contributing data to the UK State of Nature (2019) report[13] rely to a large extent on amateur contributions, but do not typically require or collect images in support of occurrence records. (See Roy and colleagues[18] for more information on the culture of citizen science in relation to species occurrence data in Britain and Ireland.)

However, in other areas, automated classification of species images using AI has further aided citizen science efforts.[19] Automated identification has made considerable progress thanks to the development of deep learning and convolutional neural networks (CNNs) in particular.[20] For example, Goëau and colleagues[21] reported on a large-scale experiment on the automatic identification of 10,000 plant species' photos (in the context of the "PlantCLEF" international challenge), resulting in impressive performances with accuracy values reaching 88%. Spanning over 5,000 categories of plants, animals, and fungi, Van Horn and colleagues[22] also reported impressive results with accuracy values higher than 81%. In Bonnet and colleagues[23] it was shown that CNNs were able to provide more accurate identifications than five out of nine specialists of the French flora who were asked to re-identify a set of plant specimens from images. Such automated identification technologies have been applied in citizen science projects to aid observers reach an identification (e.g., Pl@ntNet, www.plantnet.org; iNaturalist; Flora Incognita), but they also offer an opportunity to process large volumes of biodiversity imagery that would likely be impractical or time-inefficient for human experts to analyze.

Applications of AI to biological recording have to date typically focused on active sampling, that is, images collected specifically for the purpose of recording wildlife[24] (e.g., wildlife recording apps or camera traps). However, this has neglected large amounts of image data that are not collected for the purposes of biological recording, but which nonetheless may contain useful information about biodiversity. This includes social media imagery[25] (e.g., Flickr and Instagram), CCTV, and imagery collected along linear infrastructure (e.g., Google StreetView). These unexploited image data could be rapidly analyzed using "AI naturalists" designed to locate potential images of biodiversity and classify what they see. This is an example of internet ecology or "iEcology" as recently proposed by Jarić and colleagues,[26] whereby digital data collected for a different purpose is analyzed to gain insights into the natural world. However, these images are likely to vary in their suitability for making species identifications, the amount of metadata associated with images (e.g., is location information available?), and their temporal and spatial coverage. These issues must be explored before a reliable assessment of the utility of these untapped resources can be made.

AI naturalists, just like their human counterparts, may have their own biases which must be fully understood if the information that they generate is to be trusted and suitably utilized. For example, most AI systems can only detect or recognize already seen (or learned) objects or concepts. Benchmark datasets of images can be organized to precisely assess the limits of AI systems' ability, highlighting where human expertise is still required. Deep learning models (some of the most advanced AI algorithms) are developed with training datasets that allow them to capture discriminant visual patterns. Their performances are then strongly correlated to the quality and completeness of the datasets on which they are trained. Unbalanced, biased, or otherwise poor-quality training datasets will lead to underperforming algorithms in real conditions.[27] During the learning phases, particular attention must be given to any relevant limitations of the training data, and the gap between these and the test data on which the developed algorithms will be evaluated.[28]

We present an AI naturalist developed to create biodiversity datasets from social media image data. We use Flickr to collect images from two locations in England, one rural (Peak district) and one urban,(London) and classified images to species using the Pl@ntNet image classier. We explore the biases and challenges inherent to the image dataset and the AI classification algorithm using an expert assessment approach. Building on our

CellPress
OPEN ACCESS

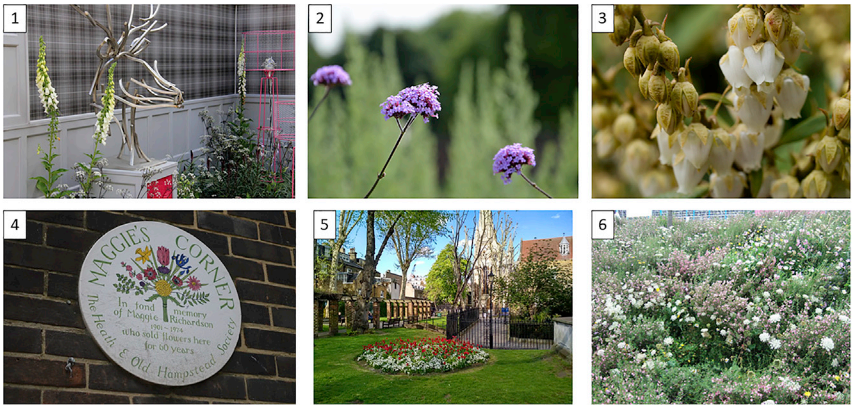

Figure 1. Randomly Selected Example Images

The top row (1–3) were all correctly identified to species by the AI classifier; 4 and 5 were classed as unidentifiable by our expert botanist, with 4 additionally classified as a representation; 6 was classed as identifiable, but as not being focused on a single species. Credits clockwise from top left: Karen Roe, "Its No Game," William Warby, "SamJKing.co.uk," Dmitry Djouce, Matt Brown (all shared under CC BY 2.0).

experience we present a checklist so that other researchers in this emerging research area can consider and avoid common pitfalls.

RESULTS

Images Are Spatially Clustered

Flickr searches returned a far greater number of images for central London (n = 55,176; 1,200 images/km^2) than for the Peak District (n = 5,486; 46 images/km^2). Images were taken between and April 26, 2003, and August 23, 2019 (Figure 1). By definition these are only the subset of images taken in these locations that had location data available. To obtain an indication of the proportion of images that did not have location data, we searched for all images that contained the word "flower" taken in the first week of July 2019, regardless of location information. July was chosen because it is the month in which Flickr records the greatest number of uploads.[29] This search returned 23,140 images, of which 25% had location information, indicating that the majority of Flickr images of flowers likely do not have location information. Heatmaps of the density of images in central London and the Peak District (Figure 2) show that the majority of images were taken around tourist sites known to be attractive because of their formal gardens. For example, in the Peak District there is a hotspot of images around Chatsworth House (Figure 2C). Images within 2 km of Chatsworth House make up 18% of all the images found in the Peak District. Images in this buffer are also more likely to be of horticultural species, when compared with images from outside the buffer (87.5% versus 51.3%, chi-square $p < 0.01$).

The Urban Area Has a Lower Proportion of Well-Classified Images

We see a clear difference between the distribution of classification scores in the rural and urban datasets (Figure 3). In London the scores have a unimodal left-skewed distribution (Dip test of multi-modality, D = 0.001, p = 0.997), while in the Peak District the distribution is bimodal with peaks near 0 and 1 (D = 0.015, $p < 0.01$). This suggests that in the urban environment there is a larger proportion of images that are either not of flowers, are not of sufficient quality, or are of species that the classifier is currently not as good at classifying.

The Rural Area Has a Higher Proportion of Images of Naturally Occurring, Native Plants

Most images from Flickr retrieved using the "flower" filter and examined by the expert (n = 1,100) had a plant as the focus of the image (81%); of these, around 79% were focused on a single species (64% of all images). Across the whole sample, approximately 83% of photos contained identifiable plant biodiversity at some taxonomic level (Figure 4). This is slightly higher than the proportion of photos explicitly focusing on plants, because in some pictures identifiable species were present even though the photographer was not considered to be targeting biodiversity. When considering images with a classification score above 0.9, the AI classifier identified 519 species in London and 184 in the Peak District.

Most of the reviewed images were of horticultural plants (73%; Figure 4), and a significant proportion were introduced by humans to their photographed location, whether in- or outdoors (70%; Figure 5A). This varied significantly between landscape settings, with shots of horticultural species, indoor plants, and introduced occurrences generally being lower in the rural setting of the Peak District than in the urban setting of London (Figures 4 and 5). This division was also clear in terms of the national native or non-native status of species, with non-native species (represented mainly by recently introduced neophytes) being more commonly photographed in London (Figure 5B), an expected finding given that shots of garden plants, which are more likely to be non-native, were also more common in London (Figure 4).

Image Composition and Subject Significantly Impact AI Classification Accuracy

The accuracy of the AI classifier, as determined by our botanical expert, increased with the AI classifier identification score, and with decreasing taxonomic resolution (Figure 6). Attributes of the photographs were also found to have an impact on whether images were likely to be correctly identified by the AI classifier and at what taxonomic level that identification was judged to be correct (Figure 7). The AI classifier performed significantly better when: images were focused on a single plant; the plant was deemed identifiable to species by the expert; the occurrence was spontaneous rather than planted; the species was not horticultural; and the plant was native. There was slightly less evidence that location (London or the Peak District) influenced the accuracy of the classifier across our samples. Attributes describing whether plants in general were the focus of the image, and whether the image was taken indoors were

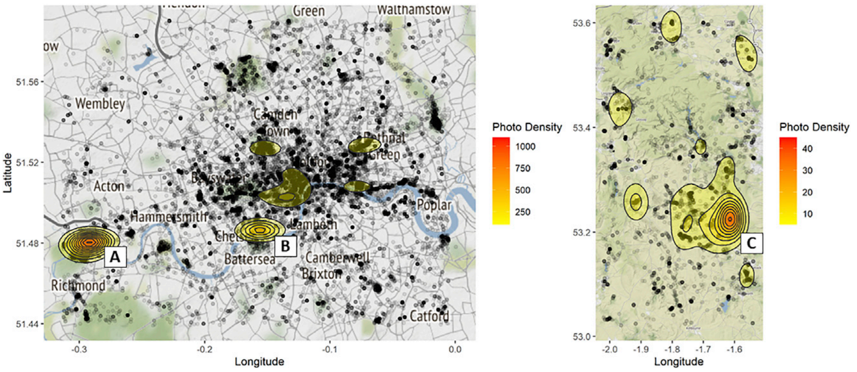

Figure 2. Spatial Distribution of Images

The spatial distribution of Flickr images returned when searching with the term "flower" in (A) London (urban) and (B) the Peak District (rural). Gray/black dots show the location of individual images. Colored areas show regions of particularly high densities of images. Hotspots correspond to: (A) Kew Gardens (a botanic garden), (B) the Chelsea Flower Show (an annual horticultural show), and (C) Chatsworth House (a large country house and gardens open to the public).

excluded from the analysis, as they correlated strongly with whether a single plant was the image focus, and whether the image was of a horticultural species, respectively. Finally, the species status category was simplified by aggregating neophytes and archaeophytes into a single "non-native" category since the number of archaeophyte records was very small (n = 7).

DISCUSSION

By combining social media APIs with AI classifiers, we were able to build an AI naturalist capable of creating biodiversity datasets from previously unexploited data sources. However, we demonstrate that there are a number of biases in the data produced, some of which may be able to be mitigated against, that must be carefully considered before the data could be used in certain types of analyses.

Image data are being collected in vast quantities all over the world, and we have looked at only one repository. We focused on Flickr because of its accessibility and rich metadata, which allowed us to filter images using text and spatial searches. Other notable sources of image data include Facebook, Twitter, Reddit, and Instagram; however, these all pose greater challenges in terms of collating and filtering, with geolocation of images perhaps being the greatest challenge. Beyond social media there are a number of other sources of data that researchers might consider, including images collected routinely from vehicles, such as train cameras, "dash-cams" in cars, and road surveys, e.g., Google StreetView.

Image licenses are of a critical interest for such research. Indeed, as it is often preferable to share images among several computational tools and infrastructure, we encourage platforms that collect images to use Creative Commons licenses where possible. This facilitates as much sharing as possible, and permits the display of images on public infrastructure for collaborative quality assessment and identification accuracy evaluation. However, if the aim is solely to generate a biological record (i.e., a piece of information relating to a taxonomic occurrence in space and time) from an online image without redistributing the image in any form, this may not infringe copyright and in some countries, such as the UK, this form of data mining is explicitly exempt from limitation by copyright.[30]

Clearly, applying AI classifiers developed for use in one situation to novel domains requires caution. Our data, retrieved using a simple filter designed to maximize the acquisition of images of plant biodiversity from Flickr, were relatively rich in photos of plant life in general, including photos that were clearly focused on single species, as might be submitted to a biodiversity identification app or tool. However, the proportion of more "scenic" or broad-focus plant shots was still high, and there was a small but significant number of indoor shots and shots of non-living representations of plants. For this reason the AI classifier accuracy we recorded is significantly

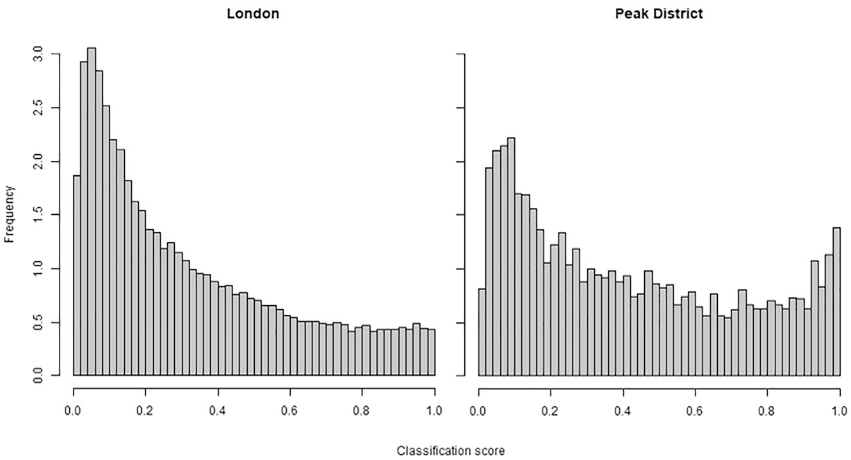

Figure 3. Distribution of Classification Scores

The distribution of classification scores assigned by the Pl@ntNet image classifier to all images from London (urban, n = 55,176) and the Peak District (rural, n = 5,486). Peak District results show a peak in the high (more confident) classification scores which is absent in the results for London.

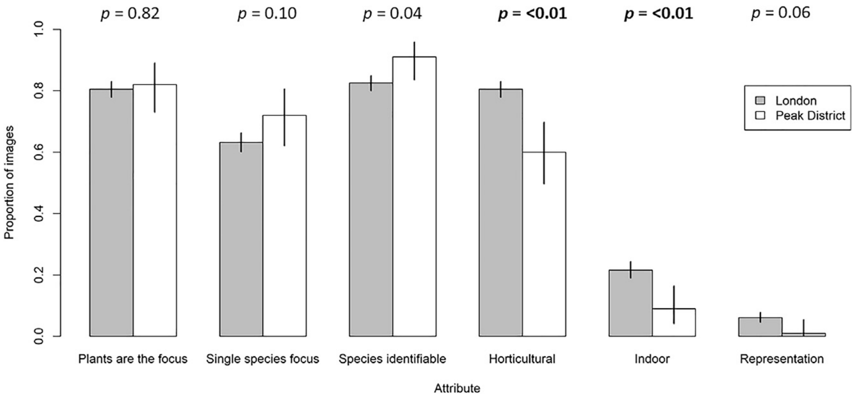

Figure 4. A Comparison of Image Attributes from London (n = 1,000) and the Peak District (n = 100)

Error bars give the 95% confidence of the proportion, p values for tests of statistical difference between proportions is given over each pair of bars. No "meta"-photos were found, therefore this category is not plotted.

lower than in other assessments of the same system.[31] This is supported by our analyses showing that the Pl@ntNet classifier was significantly less accurate with images of horticultural species (Figure 7). The impact of this so-called "open world" classification problem has been measured for plant species identification in Goëau and colleagues[32] and Joly and colleagues.[33] Moreover, the elements likely to be of most interest to biodiversity researchers, such as the representation of native or non-native established (i.e., spontaneously occurring) taxa in the dataset, were strongly context-dependent, with a far higher proportion of photos of spontaneous plants in the rural setting of the Peak District than in London (Figure 5). The London sample was dominated by human-introduced occurrences of non-natives that were likely to be garden plants, or even indoor shots, such as cut flowers or other decorations. Pilot studies, such as that presented here, are therefore likely to be essential before assuming that collections of images can be used to directly address any given question of interest, even if initial assessments of spatial coverage or tag frequency indicate a rich data source awaiting exploitation.

Previous studies based on AI classification of social multimedia data streams, have often used a small number of very general visual classes, for example, to assess ecosystem services.[34] In our case, as the main objective was to evaluate plant biodiversity, the AI classifier has to deal with a much larger number of visual classes (i.e., species). This increases the difficulty, but recent progress in automated plant species identification[35] reinforces our belief that this type of study will become easier in the years to come. Because of similar results obtained on other phyla,[3,36,37] we are confident this approach could soon be adapted for use well beyond plants, for example, to corals, fish, or birds.

Biases in our data arise in part from differences between the aims of the original data collectors (i.e., the photographers) and our aims as biodiversity researchers and ecologists. For example, the spatial distribution of our images was biased toward areas where extensive managed gardens or other displays exhibited large collections of flowering plants (Figure 2). These biases could be addressed by choosing alternative sources, changing the search terms used, or pre-filtering images. For example, choosing to collect images from social media targeted at outdoor enthusiasts (e.g., hikers), such as specialist Facebook groups or "subreddits," would be less likely to return images from formal gardens. Images may also be biased taxonomically

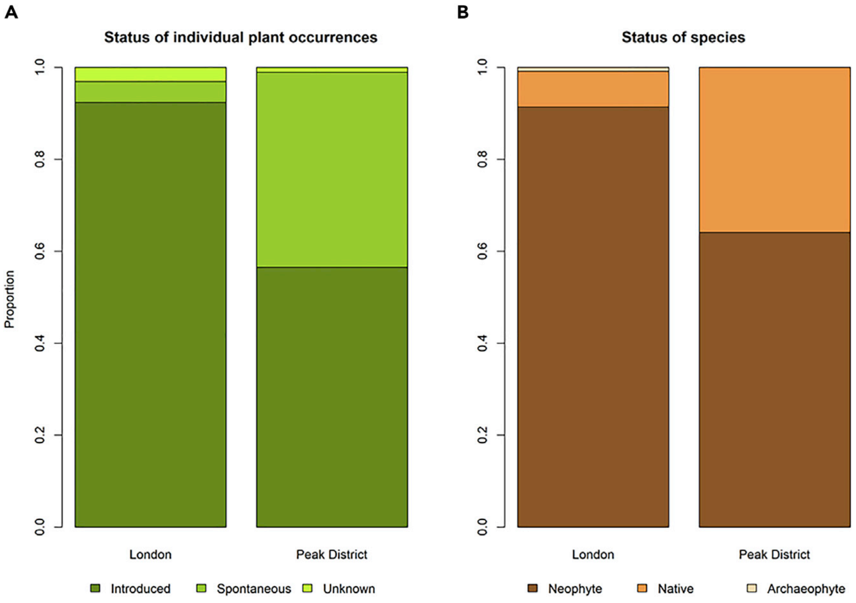

Figure 5. Status of Individual Occurrences and Species

The status of (A) individual plant occurrences and (B) species, in photographs reviewed by an expert (London: n = 1,000; Peak District: n = 100). London has both a higher proportion of introduced plant occurrences (i.e., the plant photographed has been planted or otherwise placed in its photographed location by humans), and a larger proportion of non-native species (the combination of neophytes, arrived in Britain post-1500, and archaeophytes, arrived pre-1500).

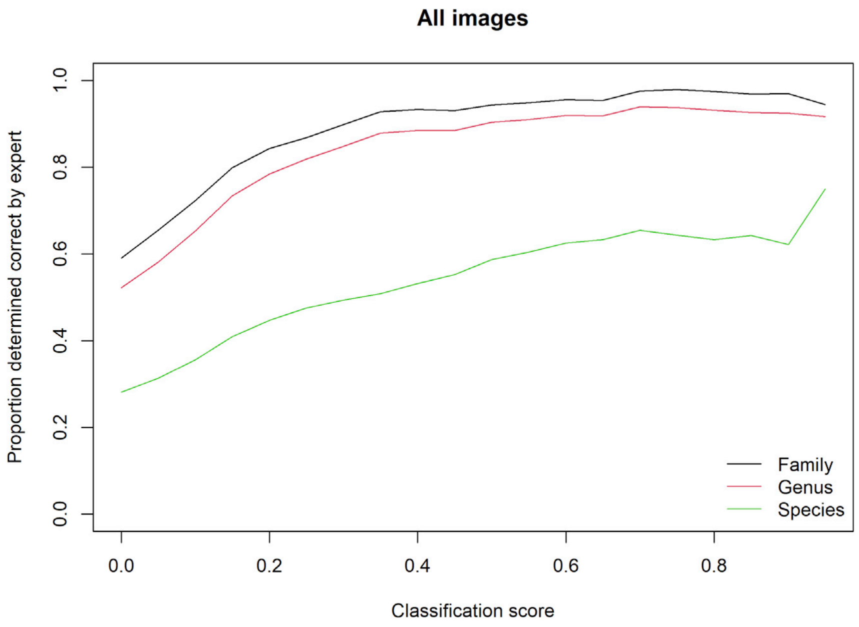

Figure 6. The Proportion of Images Deemed to Be Correctly Identified by an Expert Botanist across All Images

The proportion of images correctly identified increases with the classification score and at higher taxonomic levels. A bin width of 0.05 was used and an unsmoothed line plotted through the results.

or in terms of certain traits, for example, toward species that are typically considered more photogenic due to large colorful flowers or leaves. Search terms could be modified to either focus on a specific sub-group, e.g., searching using scientific names, or to exclude non-target images, e.g., excluding images that include the words "show" or "garden" in their metadata. Finally, high-level image classifiers could be trained to remove images that are clearly not plants, for example, removing images of animals, paintings. High-level classifiers developed to separate images that contain plants from those that do not, without looking to identify species, could be used to find images worthy of further examination in large datasets that do not have metadata (such as titles and descriptions), removing the need for keyword searches, such as that used in this study.

Even if the traditional questions of ecology or conservation biology concerning factors determining species' distributions and abundances cannot be directly addressed by harvested social media imagery, this does not necessarily mean that these data have no value for broader questions of environmental or socio-cultural interest: biases are only biases in relation to some specified research aim. Thinking more broadly, collections of plant photos contain information about the preferences of individuals for different species, preferences for formal versus more naturalistic gardens, and on seasonal patterns of human activity. This approach could therefore be a tool in the domain of "conservation culturomics,"[38] which uses quantitative analyses to explore changes in human behavior in conservation science. These preferences have been used previously to map multiple recreational beneficiaries,[39] detect human activity patterns,[40] or to quantify the attractiveness of outdoor areas.[41,42] Trends in submitted images across years could also be of interest; for example, these could indicate changing levels of interest in wilder forms of gardening or park maintenance that are likely to be of interest to conservationists or those quantifying ecosystem services; photographs of plant-pollinator interactions could illustrate trends in public interest in potential insect declines; increases in images of non-native species could indicate increased awareness of invasive non-native species. These topics are all suggested as possible uses of Flickr data based on our sampled assessment.

Once data are generated by AI naturalists we must consider how they are shared to ensure recognition of their authors, observation traceability, and long-term accessibility. We suggest following the TDWG standards,[43] such as Darwin-Core among others, to ensure the data are interoperable with other biodiversity datasets and can be shared via the GBIF portal (www.gbif.org). Metadata accompanying these datasets should include the AI model architecture, technical specificities of the AI model training phase, digital object identifier of the dataset used to train it, AI model version, classification score of each image, date, location, photograph name, and image license. Classification score, which provides a mathematical value of the confidence in the prediction of a model for a specific image, is particularly important as it can be used in subsequent analyses to filter the data by the level of confidence in the classification accuracy. A researcher will need to define the appropriate balance to choose the threshold classification score at which to filter these data according to the research question being addressed, as was done in a study of invasive species by Botella and colleagues.[44] An AI classified dataset from Pl@antNet has already been published on GBIF,[45] illustrating the interest of the scientific community in this new type of data. Generalization of our approach to larger geographical scale and other social networks could open the door to a much larger number of automatically identified biodiversity observations on this and other platforms.

For any given research question, ecologists and data scientists should carefully consider the steps that might be required to ensure the relevance and accuracy of AI-generated data for any given research question. To aid this we have summarized our experience into an eight-point list of questions which we recommend researchers ask themselves when using AI classifier naturalists:

1. Does the spatial distribution of images fit your needs? Images from social media are often aggregated in areas of high population density or tourist hotspots. If the distribution is biased in some way, could this be accounted for in subsequent analyses?
2. Can you filter images before classification? For example, filtering can be done by carefully selecting your source

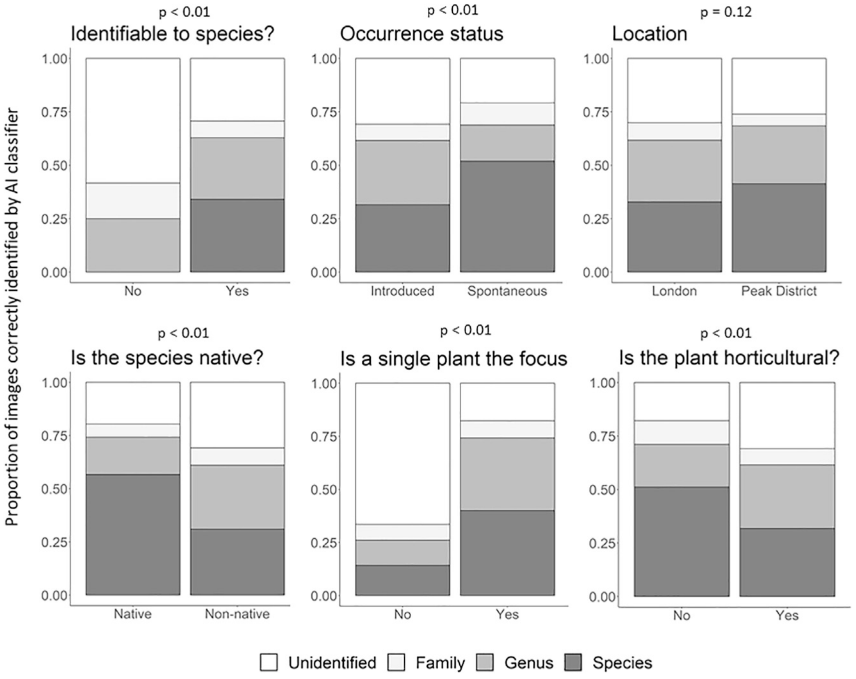

Figure 7. Expert Assessment of AI Classifier Accuracy

Panels show the impact of different attributes on the accuracy of the AI classifier. p values for tests of these relationships using univariable ordinal logistic regressions are given above each panel. Specifically, the p value here is the model-based probability that the ordinal-dependent variable distributions arise from the same set of latent cutpoints between the two levels of each independent variable attribute. Unidentified, image judged to be incorrectly classified by the AI classifier at the family level (and therefore also at all nested taxonomic levels); Family, image judged to be correctly classified at the family level, but not at nested levels; Genus, image judged to be correctly classified at the genus level, but not at nested levels; Species, image judged to be correctly classified to the species level.

of images, using GPS location, focusing on keywords in image metadata, or using high-level AI classifiers to remove non-target images.

3. What is the appropriate taxonomic resolution for your study? This will be driven by your research question, as well as an assessment of the AI naturalist's accuracy. Classifiers will tend to be more accurate at higher taxonomic levels, but this may vary between taxonomic groups.
4. What reporting biases exist in your dataset? For example, to what degree are charismatic species over-represented, or nocturnal species under-represented? Can you filter the data, or model the results to account for these biases if they are relevant?
5. Do reporting biases change over space or time? We observed significant differences in reporting bias between urban and rural settings, and we anticipate that temporal biases are likely to exist where public interest in elements of the natural environment change over time.
6. How will you propagate uncertainty in classifications? AI classifications are associated with a classification score which is indicative of the uncertainty in the identification. This can be used both as a threshold for removing erroneous results, and/or could be included in models to account for variation in uncertainty between observations.
7. Is the dataset used to train your AI naturalist a good match to the images being classified? A poor match between training and prediction datasets will result in higher error rates, which may not always be associated with low classification scores.
8. Have you adequately documented your dataset? To ensure reproducibility and interoperability ensure that you document the model used for classification, filtering steps used to collate images, and other metadata useful to future researchers, and which may be specified in data standards for AI-generated biodiversity which do not exist at the time of writing.

EXPERIMENTAL PROCEDURES

Resource Availability

Lead Contact

Further information and requests for resources should be directed to and will be fulfilled by the Lead Contact, Tom A. August (tomaug@ceh.ac.uk).

Materials Availability

This study did not generate new unique materials or reagents.

Data and Code Availability

The published article includes datasets and code generated and analyzed during this study in the supplementary materials.

Methods

Searching Flickr

We accessed publicly visible image data on the website Flickr (www.flickr.com). Flickr is a website used for image hosting and has an application programming interface (API) that allows queries of the image database. Images hosted on Flickr tend to be better annotated with tags, location, and description than other potential sources of image data, such as Twitter, potentially because Flickr is targeted at people with a keen interest in photography. This potentially explains why Flickr has been used in previous studies to develop a better understanding of people's subjective experience of the environment in which they live,[41] and to automate assessment of cultural ecosystem services.[34] We searched Flickr using the R-package "photosearcher"[46] (https://github.com/ropensci/photosearcher) for images that contained the word "flower" in either their title, description, or tags. We found that this search term resulted in the best balance between the quantity and quality of images returned when compared with other search terms, such as "plant," or filtering using only mentions in image tags. We found few images specified the Latin or common name in the queryable metadata, therefore queries based on taxonomic lists would be unlikely to return many images (extensive taxonomic labeling would also imply that AI identification would potentially

be unnecessary). We searched for these images in two locations: London (mainly urban; bounding box = −0.312836, 51.439050, −0.005219, 51.590237; area = 46 km^2), and the Peak District (mainly rural; bounding box = −2.021484, 53.019740, −1.533966, 53.603914; area = 119 km^2).

AI Classification

Flickr images were classified using a deep learning-based classifier trained on Pl@ntNet data. Pl@ntNet is a participatory research and educational platform for the production, aggregation, and dissemination of botanical observations.[47,48] Initiated in 2009, it relies on a web and mobile infrastructure to support the identification of plants by AI classification. It covers a significant part of the European and North American flora, and an increasing number of species in tropical regions. Images are classified by a CNN that is periodically trained in a supervised manner on the valid plant observations produced and revised by the Pl@ntNet user community (currently 1.8 million user accounts). At the time of writing, the CNN architecture used is the inception model[49] extended with batch normalization.[50] The network is pre-trained on the commonly used ImageNet dataset and fine-tuned on Pl@ntNet data. Pl@ntNet currently covers 30,261 species illustrated by more than 2.9 million images. The taxonomic coverage of our study is therefore one to three orders of magnitude larger than previously published studies making use of automated species identification for ecological research. The training of Pl@ntNet CNN requires the mobilization of a high-performance computing infrastructure and expertise in deep, distributed, and large-scale learning. Thus, the resulting classification tool is in itself a major advance in biodiversity data science.

Access to the Pl@ntNet classification tool is provided through a dedicated API available at my.plantnet.org. The main feature of this API is a RESTful JSON-based web service that can accept one to five images of a plant and returns a list of likely species. The species are associated with classification scores (the softmax output of the CNN), as well as a list of matching images retrieved from the database. To facilitate the implementation of future studies based on the methodology of this paper, we have developed the "plantnet"[51] R-package allowing users to query the Pl@ntNet API. The package is available online at https://github.com/BiologicalRecordsCentre/plantnet.

Flickr images were submitted one-by-one to the API, and only the taxonomic identification associated with the highest classification score was retained for each image. No thresholding on the classification score was applied. Only the classification scores and image metadata were stored, Flickr images were not downloaded. A demonstration of the workflow, utilizing the photosearcher[46] and plantnet[51] R-packages, is given in the Supplemental Information.

Expert Assessment

Flickr image URLs, metadata (e.g., geolocations), and Pl@ntNet classification information were stored in CSV files (see Data S1). For each area—London/Urban or the Peak District/Rural—1,000 (1.8%) and 100 (1.8%) random image samples were taken, respectively. An expert botanist (OP, author) subsequently assessed each image, along with its location, other relevant metadata, such as the image title, and the Pl@ntNet prediction, in a web browser using a custom RShiny app (see Supplemental Information). The original Flickr URL of each image was also provided so that the expert could view other contextual information, such as comments on the photo and adjacent images taken by the same photographer. Within the app, the expert assessed each photo using a standard set of questions. These were: (1) whether real plants were the main focus of the photo; (2) if so, whether a single plant species was the focus; (3) whether any real plant in a photo was considered to be clearly identifiable to species; (4) whether the Pl@ntNet identification was considered correct at each of the family, genus, and species levels; (5) the national (British) status of the focal species of the image, i.e., whether the species was native to Britain, or considered to have been introduced by humans, either recently (post-1500; a "neophyte") or anciently (pre-1500; an "archaeophyte"); (6) whether the occurrence of the focal species in the photograph was spontaneous (i.e., naturally occurring), introduced (i.e., the occurrence was the responsibility of a human planting or placing the species in its photographed location), or unknown (e.g., the photograph was such an extreme close-up, and the species is known to be both present in gardens and the wild, such that the decision between spontaneous and introduced cannot be deduced from the photo with any certainty); (7) whether the plant is widely used in horticulture; (8) whether the photo was taken indoors; (9) whether the photo is actually of a representation of a plant rather than a real plant (e.g., a sculpture, embroidery, silk flower); and (10) whether the photo was a picture of another photo of a real plant (i.e., a "meta"-photo). Example images are shown in Figure 1. The effect of these attributes on the ability of the AI to correctly classify the image at different taxonomic levels was tested by a series of univariable ordinal logistic regressions using the polr function in the R-package MASS.[52]

SUPPLEMENTAL INFORMATION

Supplemental Information can be found online at https://doi.org/10.1016/j.patter.2020.100116.

ACKNOWLEDGMENTS

An early demonstration of this concept was developed at the British Ecological Society Quantitative Ecology Special Interest Group hackathon, led by T.A. Thanks to members of the hackathon team for their contributions: Nathan Fox, Celia Marlowe, Joseph Millard, Nadia Bystriakova, Elliot Shayle, and Roy Sanderson. T.A. was supported by COST action CA17122 "Increasing understanding of alien species through citizen science" and Natural Environment Research Council award number NE/R016429/1 as part of the UK-SCAPE program delivering National Capability. We thank three anonymous reviewers for their comments.

AUTHOR CONTRIBUTIONS

T.A. and O.P. conceived the ideas. O.P. undertook the expert assessment. T.A. undertook the analyses. P.B. and A.J. facilitated access to the Pl@ntNet API. All authors contributed equally to the writing of the manuscript.

DECLARATION OF INTERESTS

The authors declare no competing interests.

Received: May 29, 2020
Revised: August 4, 2020
Accepted: September 7, 2020
Published: October 9, 2020

REFERENCES

1. Schroeck, M., Shockley, R., Smart, J., Romero-Morales, D., and Tufano, P. (2012). Analytics: The Real-World Use of Big Data (IBM Institute for Business Value).
2. Sun, A.Y., and Scanlon, B.R. (2019). How can Big Data and machine learning benefit environment and water management: a survey of methods, applications, and future directions. Environ. Res. Lett. *14*, 073001.
3. Tabak, M.A., Norouzzadeh, M.S., Wolfson, D.W., Sweeney, S.J., Vercauteren, K.C., Snow, N.P., Halseth, J.M., Salvo, P.A.D., Lewis, J.S., White, M.D., et al. (2019). Machine learning to classify animal species in camera trap images: applications in ecology. Methods Ecol. Evol. *10*, 585–590.
4. Gibb, R., Browning, E., Glover-Kapfer, P., and Jones, K.E. (2019). Emerging opportunities and challenges for passive acoustics in ecological assessment and monitoring. Methods Ecol. Evol. *10*, 169–185.
5. Efron, B., and Hastie, T. (2016). Computer Age Statistical Inference (Cambridge University Press).
6. Lintott, C.J., Schawinski, K., Slosar, A., Land, K., Bamford, S., Thomas, D., Raddick, M.J., Nichol, R.C., Szalay, A., Andreescu, D., et al. (2008). Galaxy zoo: morphologies derived from visual inspection of galaxies from the Sloan digital sky survey. Mon. Not. R. Astron. Soc. *389*, 1179–1189.
7. Nguyen, K., Fookes, C., Jillela, R., Sridharan, S., and Ross, A. (2017). Long range iris recognition: a survey. Pattern Recognit. *72*, 123–143.
8. Zhang, Y., Li, X., Gao, L., and Li, P. (2018). A new subset based deep feature learning method for intelligent fault diagnosis of bearing. Expert Syst. Appl. *110*, 125–142.
9. Rattani, A., and Derakhshani, R. (2018). A survey of mobile face biometrics. Comput. Electr. Eng. *72*, 39–52.

CellPress
OPEN ACCESS

10. Wang, A. (2006). The Shazam music recognition service. Commun. ACM *49*, 44–48.

11. CBD (2014). Global Biodiversity Outlook: A Mid-term Assessment of Progress towards the Implementation of the Strategic Plan for Biodiversity 2011–2020 (Secretariat of the Convention on Biological Diversity).

12. Prip, C. (2018). The Convention on Biological Diversity as a legal framework for safeguarding ecosystem services. Ecosyst. Serv. *29*, 199–204.

13. Hayhow, D.B., Burns, F., Eaton, M.A., Al Fulaij, N., August, T.A., Babey, L., Bacon, L., Bingham, C., Boswell, J., Boughey, K.L., et al. (2016). State of Nature 2016. http://www.rspb.org.uk/Images/State%20of%20Nature%20UK%20report_%2020%20Sept_tcm9-424984.pdf.

14. Preston, C.D. (2013). Following the BSBI's lead: the influence of the Atlas of the British Flora, 1962–2012. New J. Bot. *3*, 2–14.

15. Pescott, O.L., Walker, K.J., Pocock, M.J.O., Jitlal, M., Outhwaite, C.L., Cheffings, C.M., Harris, F., and Roy, D.B. (2015). Ecological monitoring with citizen science: the design and implementation of schemes for recording plants in Britain and Ireland. Biol. J. Linn. Soc. *115*, 505–521.

16. Silvertown, J. (2009). A new dawn for citizen science. Trends Ecol. Evol. *24*, 467–471.

17. Silvertown, J., Harvey, M., Greenwood, R., Dodd, M., Rosewell, J., Rebelo, T., Ansine, J., and McConway, K. (2015). Crowdsourcing the identification of organisms: a case-study of iSpot. ZooKeys *480*, 125–146.

18. Roy, H.E., Preston, C.D., and Roy, D.B. (2015). Fifty years of the biological records centre. Biol. J. Linn. Soc. *115*, 469–474.

19. Ceccaroni, L., Bibby, J., Roger, E., Flemons, P., Michael, K., Fagan, L., and Oliver, J.L. (2019). Opportunities and risks for citizen science in the age of artificial intelligence. Citiz. Sci. Theor. Pract. *4*, 29.

20. Wäldchen, J., and Mäder, P. (2018). Machine learning for image based species identification. Methods Ecol. Evol. *9*, 2216–2225.

21. Goëau, H., Bonnet, P., and Joly, A. Overview of ExpertLifeCLEF 2018: how far automated identification systems are from the best experts? CLEF - Conference and Labs of the Evaluation Forum, September 2018, Avignon, France (hal-01913244).

22. Van Horn, G., Mac Aodha, O., Song, Y., Cui, Y., Sun, C., Shepard, A., Adam, H., Perona, P., and Belongie, S. (2018). The iNaturalist species classification and detection dataset. In 2018 IEEE/CVF Conference on Computer Vision and Pattern Recognition (IEEE), pp. 8769–8778.

23. Bonnet, P., Goëau, H., Hang, S.T., Lasseck, M., Šulc, M., Malécot, V., Jauzein, P., Melet, J.-C., You, C., and Joly, A. (2018). Plant identification: experts vs. machines in the era of deep learning. In Multimedia Tools and Applications for Environmental & Biodiversity Informatics Multimedia Systems and Applications, A. Joly, S. Vrochidis, K. Karatzas, A. Karppinen, and P. Bonnet, eds. (Springer), pp. 131–149.

24. Norouzzadeh, M.S., Nguyen, A., Kosmala, M., Swanson, A., Palmer, M.S., Packer, C., and Clune, J. (2018). Automatically identifying, counting, and describing wild animals in camera-trap images with deep learning. Proc. Natl. Acad. Sci. U S A *115*, E5716–E5725.

25. Di Minin, E., Fink, C., Tenkanen, H., and Hiippala, T. (2018). Machine learning for tracking illegal wildlife trade on social media. Nat. Ecol. Evol. *2*, 406–407.

26. Jarić, I., Correia, R.A., Brook, B.W., Buettel, J.C., Courchamp, F., Di Minin, E., Firth, J.A., Gaston, K.J., Jepson, P., Kalinkat, G., et al. (2020). iEcology: harnessing large online resources to generate ecological insights. Trends Ecol. Evol. *35*, 630–639..

27. Carranza-Rojas, J., Mata-Montero, E., and Goeau, H. (2018). Hidden biases in automated image-based plant identification. In 2018 IEEE International Work Conference on Bioinspired Intelligence (IWOBI) (IEEE), pp. 1–9.

28. Ho, S.Y., Wong, L., and Goh, W.W.B. (2020). Avoid oversimplifications in machine learning: going beyond the class-prediction accuracy. Patterns *1*, 100025.

29. Michel, F. (2012). Flickr may have lost 63% of its photos after being acquired by SmugMug. https://www.flickr.com/photos/franckmichel/6855169886.

30. (2012). The Copyright and Rights in Performances (Research, Education, Libraries and Archives) Regulations 2014. https://www.legislation.gov.uk/uksi/2014/1372/regulation/3/made.

31. Goëau, H., Bonnet, P., and Joly, A. (2017). Plant identification based on noisy web data: the amazing performance of deep learning (LifeCLEF 2017). CLEF: Conference and Labs of the Evaluation Forum.

32. Goëau, H., Bonnet, P., and Joly, A. (2016). Plant Identification in an Open-world (LifeCLEF 2016). CLEF: Conference and Labs of the Evaluation Forum. https://hal.archives-ouvertes.fr/hal-01373780/document.

33. Joly, A., Goëau, H., Champ, J., Dufour-Kowalski, S., Müller, H., and Bonnet, P. (2016). Crowdsourcing biodiversity monitoring: how sharing your photo stream can sustain our planet. In MM '16: Proceedings of the 24th ACM International Conference on Multimedia (ACM), pp. 958–967.

34. Richards, D.R., and Tunçer, B. (2018). Using image recognition to automate assessment of cultural ecosystem services from social media photographs. Ecosyst. Serv. *31*, 318–325.

35. Wäldchen, J., Rzanny, M., Seeland, M., and Mäder, P. (2018). Automated plant species identification—trends and future directions. PLoS Comput. Biol. *14*, e1005993.

36. Marini, S., Corgnati, L., Mantovani, C., Bastianini, M., Ottaviani, E., Fanelli, E., Aguzzi, J., Griffa, A., and Poulain, P.-M. (2018). Automated estimate of fish abundance through the autonomous imaging device GUARD1. Measurement *126*, 72–75.

37. Villon, S., Mouillot, D., Chaumont, M., Darling, E.S., Subsol, G., Claverie, T., and Villéger, S. (2018). A deep learning method for accurate and fast identification of coral reef fishes in underwater images. Ecol. Inform. *48*, 238–244.

38. Ladle, R.J., Correia, R.A., Do, Y., Joo, G.-J., Malhado, A.C., Proulx, R., Roberge, J.-M., and Jepson, P. (2016). Conservation culturomics. Front. Ecol. Environ. *14*, 269–275.

39. Gosal, A.S., Geijzendorffer, I.R., Václavík, T., Poulin, B., and Ziv, G. (2019). Using social media, machine learning and natural language processing to map multiple recreational beneficiaries. Ecosyst. Serv. *38*, 100958.

40. Koylu, C., Zhao, C., and Shao, W. (2019). Deep neural networks and kernel density estimation for detecting human activity patterns from geo-tagged images: a case study of birdwatching on Flickr. ISPRS Int. J. Geo inf. *8*, 45.

41. Seresinhe, C.I., Moat, H.S., and Preis, T. (2017). Quantifying scenic areas using crowdsourced data. Environ. Plan. B Urban Anal. City Sci. https://doi.org/10.1177/0265813516687302.

42. Seresinhe, C.I., Preis, T., and Moat, H.S. (2017). Using deep learning to quantify the beauty of outdoor places. R. Soc. Open Sci. *4*, 170170.

43. Berendsohn, W., Güntsch, A., Hoffmann, N., Kohlbecker, A., Luther, K., and Müller, A. (2011). Biodiversity information platforms: from standards to interoperability. ZooKeys *150*, 71–87.

44. Botella, C., Joly, A., Bonnet, P., Monestiez, P., and Munoz, F. (2018). Species distribution modeling based on the automated identification of citizen observations. Appl. Plant Sci. *6*, e1029.

45. Affouard, A., Joly, A., and Bonnet, P.. Pl@ntNet automatically identified occurrences, accessed via GBIF.org on 2020-07-27 https://doi.org/10.15468/mma2ec.

46. Fox, N., Mancini, F., Graham, L., Sutter, L., and August, T. (2020). Photosearcher 1.0. https://docs.ropensci.org/photosearcher/.

47. Joly, A., Bonnet, P., Goëau, H., Barbe, J., Selmi, S., Champ, J., Dufour-Kowalski, S., Affouard, A., Carré, J., Molino, J.-F., et al. (2016). A look inside the Pl@ntNet experience. Multimed. Syst. *22*, 751–766.

48. Affouard, A., Goeau, H., Bonnet, P., Lombardo, J.-C., and Joly, A. (2017). Pl@ntNet App in the Era of Deep Learning, p. 6.

49. Szegedy, C., Vanhoucke, V., Ioffe, S., Shlens, J., and Wojna, Z. (2016). Rethinking the inception architecture for computer vision. In 2016 IEEE

Conference on Computer Vision and Pattern Recognition (CVPR) (IEEE), pp. 2818–2826.

50. Ioffe, S., and Szegedy, C. (2015). Batch normalization: accelerating deep network training by reducing internal covariate shift. arXiv, 1502.03167.

51. August, T. (2019). Plantnet: automated plant identification with PlantNet 0.1.0. https://github.com/BiologicalRecordsCentre/plantnet.

52. Venables, W.N., and Ripley, B.D. (2002). Modern Applied Statistics with S, Fourth Edition (Springer-Verlag).

PATTER, Volume *1*

Supplemental Information

AI Naturalists Might Hold the Key to Unlocking Biodiversity Data in Social Media Imagery

Tom A. August, Oliver L. Pescott, Alexis Joly, and Pierre Bonnet

Accessing and Identifying Images

Introduction

In this document we outline the methods used to collate images from Flickr, and analyse images using Pl@ntNet, used in the paper "August et al. 2020. AI naturalists might hold the key to unlocking biodiversity data in social media imagery. Patterns".

This code heavily relies on two R packages, `photosearcher` and `plantnet`. Here we show the methods used in the paper as well as some simpler cases to allow demonstration. It is important to note that with the ever changing data on Flickr and the ever changing model behind the Pl@ntNet API, it will not be possible to exactly replicate our analysis even if using the same code.

Accessing images

Images were accessed using the `photosearcher` package. At the time of writing this is available at: https://github.com/ropensci/photosearcher, and is accompanied by tutorials and help documentation. We performed two searches with this function, one for each of the target survey areas. These searches can take some time to run and require the user to first set up their user tokens, described in the package help documentation.

```
# Get the image data for London
photo_meta1 <- photo_search(mindate_taken = "2000-03-01",
                            maxdate_taken = "2019-10-01",
                            text = "flower",
                            bbox = "-2.021484,53.019740,-1.533966,53.603914")

# Get the image data for the Peak District
photo_meta2 <- photo_search(mindate_taken = "2000-03-01",
                            maxdate_taken = "2019-10-01",
                            text = "flower",
                            bbox = "-0.312836,51.439050,-0.005219,51.590237")
```

For demonstration purposes here is a small example for the London data on a single day

```
# Install the package from GitHub
devtools::install_github("ropensci/photosearcher")

## Skipping install of 'photosearcher' from a github remote, the SHA1
(65a9f488) has not changed since last install.
##   Use `force = TRUE` to force installation

library(photosearcher)

# Get the image data for London
demo <- photo_search(mindate_taken = "2019-10-01",
```

```
                     maxdate_taken = "2019-10-30",
                     text = "flower",
                     bbox = "-2.021484,53.019740,-1.533966,53.603914")
str(demo)

## 'data.frame':    5 obs. of  62 variables:
##  $ license              : int  0 0 0 0 1
##  $ id                   : num  4.89e+10 4.88e+10 4.88e+10 4.89e+10 4.89e+10
##  $ owner                : chr  "16819123@N06" "16819123@N06" "16819123@N06"
"16819123@N06" ...
##  $ secret               : chr  "578f9e80f7" "457b740e9d" "d16cbec8b0"
"2971847ee0" ...
##  $ server               : num  65535 65535 65535 65535 65535
##  $ farm                 : num  66 66 66 66 66
##  $ title                : chr  "OrangeGlow" "Sunlit" "Wall" "Contemplation"
...
##  $ ispublic             : logi  NA NA NA NA NA
##  $ isfriend             : logi  NA NA NA NA NA
##  $ isfamily             : logi  NA NA NA NA NA
##  $ dateupload           : POSIXct, format: "2019-10-13 11:49:38" "2019-10-
03 11:56:09" ...
##  $ lastupdate           : POSIXct, format: "2020-04-06 23:51:03" "2019-11-
03 17:16:17" ...
##  $ datetaken            : POSIXct, format: "2019-10-02 13:49:21" "2019-10-
02 13:52:23" ...
##  $ datetakengranularity: int  0 0 0 0 0
##  $ datetakenunknown     : int  0 0 0 0 0
##  $ count_views          : int  759 740 764 1280 168
##  $ count_faves          : int  23 14 17 8 1
##  $ count_comments       : int  3 1 0 0 0
##  $ tags                 : chr  "nikon d7100 sigma 70mm orange orton
ortoneffect flowers surreal meerbrook staffs staffordshire abstract" "niko
d7100 sigma 70mm flower orange chiaroscuro churchyard grindon staffs
staffordshire" "nikon d7100 nikkor 55300mm wall whitewash flowers
hangingbasket window grindon staffs staffordshire" "nikon d600 nikkor 50mm
f18g flowers lily lilies whiteflowers stilllife church longnor staffs
staffordshire" ...
##  $ latitude             : num  53.1 53.1 53.1 53.2 53.2
##  $ longitude            : num  -2.02 -1.87 -1.87 -1.87 -1.63
##  $ accuracy             : int  16 16 15 16 16
##  $ context              : int  0 0 0 0 0
##  $ place_id             : chr  "" "" "" "" ...
##  $ woeid                : num  2642828 2647866 2647866 2643650 2650241
##  $ geo_is_public        : logi  NA NA NA NA NA
##  $ geo_is_contact       : logi  NA NA NA NA NA
##  $ geo_is_friend        : logi  NA NA NA NA NA
##  $ geo_is_family        : logi  NA NA NA NA NA
##  $ url_sq               : chr
"https://live.staticflickr.com/65535/48890416561_578f9e80f7_s.jpg"
"https://live.staticflickr.com/65535/48836184833_457b740e9d_s.jpg"
```

```
"https://live.staticflickr.com/65535/48836214616_d16cbec8b0_s.jpg"
"https://live.staticflickr.com/65535/48929255968_2971847ee0_s.jpg" ...
##  $ height_sq           : num  75 75 75 75 75
##  $ width_sq            : num  75 75 75 75 75
##  $ url_t               : chr
"https://live.staticflickr.com/65535/48890416561_578f9e80f7_t.jpg"
"https://live.staticflickr.com/65535/48836184833_457b740e9d_t.jpg"
"https://live.staticflickr.com/65535/48836214616_d16cbec8b0_t.jpg"
"https://live.staticflickr.com/65535/48929255968_2971847ee0_t.jpg" ...
##  $ height_t            : num  67 67 67 67 67
##  $ width_t             : num  100 100 100 100 100
##  $ url_s               : chr
"https://live.staticflickr.com/65535/48890416561_578f9e80f7_m.jpg"
"https://live.staticflickr.com/65535/48836184833_457b740e9d_m.jpg"
"https://live.staticflickr.com/65535/48836214616_d16cbec8b0_m.jpg"
"https://live.staticflickr.com/65535/48929255968_2971847ee0_m.jpg" ...
##  $ height_s            : num  160 160 160 160 160
##  $ width_s             : num  240 240 240 240 240
##  $ url_q               : chr
"https://live.staticflickr.com/65535/48890416561_578f9e80f7_q.jpg"
"https://live.staticflickr.com/65535/48836184833_457b740e9d_q.jpg"
"https://live.staticflickr.com/65535/48836214616_d16cbec8b0_q.jpg"
"https://live.staticflickr.com/65535/48929255968_2971847ee0_q.jpg" ...
##  $ height_q            : num  150 150 150 150 150
##  $ width_q             : num  150 150 150 150 150
##  $ url_m               : chr
"https://live.staticflickr.com/65535/48890416561_578f9e80f7.jpg"
"https://live.staticflickr.com/65535/48836184833_457b740e9d.jpg"
"https://live.staticflickr.com/65535/48836214616_d16cbec8b0.jpg"
"https://live.staticflickr.com/65535/48929255968_2971847ee0.jpg" ...
##  $ height_m            : num  333 333 333 334 333
##  $ width_m             : num  500 500 500 500 500
##  $ url_n               : chr
"https://live.staticflickr.com/65535/48890416561_578f9e80f7_n.jpg"
"https://live.staticflickr.com/65535/48836184833_457b740e9d_n.jpg"
"https://live.staticflickr.com/65535/48836214616_d16cbec8b0_n.jpg"
"https://live.staticflickr.com/65535/48929255968_2971847ee0_n.jpg" ...
##  $ height_n            : num  213 213 213 214 213
##  $ width_n             : num  320 320 320 320 320
##  $ url_z               : chr
"https://live.staticflickr.com/65535/48890416561_578f9e80f7_z.jpg"
"https://live.staticflickr.com/65535/48836184833_457b740e9d_z.jpg"
"https://live.staticflickr.com/65535/48836214616_d16cbec8b0_z.jpg"
"https://live.staticflickr.com/65535/48929255968_2971847ee0_z.jpg" ...
##  $ height_z            : num  427 427 427 427 427
##  $ width_z             : num  640 640 640 640 640
##  $ url_c               : chr
"https://live.staticflickr.com/65535/48890416561_578f9e80f7_c.jpg"
"https://live.staticflickr.com/65535/48836184833_457b740e9d_c.jpg"
"https://live.staticflickr.com/65535/48836214616_d16cbec8b0_c.jpg"
```

```
"https://live.staticflickr.com/65535/48929255968_2971847ee0_c.jpg" ...
##  $ height_c           : num  533 533 533 534 533
##  $ width_c            : num  800 800 800 800 800
##  $ url_l              : chr
"https://live.staticflickr.com/65535/48890416561_578f9e80f7_b.jpg"
"https://live.staticflickr.com/65535/48836184833_457b740e9d_b.jpg"
"https://live.staticflickr.com/65535/48836214616_d16cbec8b0_b.jpg"
"https://live.staticflickr.com/65535/48929255968_2971847ee0_b.jpg" ...
##  $ height_l           : num  683 683 683 684 683
##  $ width_l            : num  1024 1024 1024 1024 1024
##  $ url_o              : chr  NA NA NA NA ...
##  $ height_o           : num  NA NA NA NA 3648
##  $ width_o            : num  NA NA NA NA 5472
##  $ description        : chr  "An Orton effect image, achieved by
subtracting 30% of the original from a very blurred version.\nIn the
churchy"| __truncated__ "Flowers starkly picked out by a shaft of sunlight in
the churchyard at Grindon, Staffordshire.\nCamera set at -"| __truncated__
"The wall of house in Grindon, Staffordshire" "Lilies in the church at
Longnor, Staffordshire" ...
##  $ license_name       : chr  "All Rights Reserved" "All Rights Reserved"
"All Rights Reserved" "All Rights Reserved" ...
##  $ license_url        : chr  "NA" "NA" "NA" "NA" ...
```

As you can see a large amount of metadata is returned including links to the images at various sizes.

Image identification

Once you have the URLs for the images of interest these can be passed to the `plantnet` package for classification. The `plantnet` package is available on GitHub and has tutorials and help documentation - https://github.com/BiologicalRecordsCentre/plantnet

To use this API you need to create a key on the Pl@ntNet API pages - https://my.plantnet.org/

```
# Install the package from GitHub
devtools::install_github("BiologicalRecordsCentre/plantnet")

## Skipping install of 'plantnet' from a github remote, the SHA1 (260601c7)
has not changed since last install.
##   Use `force = TRUE` to force installation

library(plantnet)

##
## Attaching package: 'plantnet'

## The following object is masked from 'package:graphics':
##
##     identify
```

```r
# Lets take a look at one of the images
download.file(demo$url_l[4],
              'temp.jpg', mode = 'wb')
jj <- readJPEG('temp.jpg', native = TRUE)
plot(0:1, 0:1, type = "n", ann = FALSE, axes = FALSE)
rasterImage(jj,0,0,1,1)
```

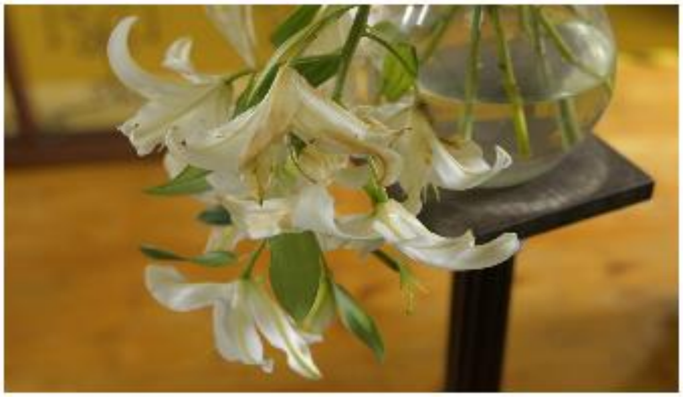

```r
# Now lets identify this image
id <- identify(key = Secret_Key,
               imageURL = demo$url_l[4])
id

##       score     latin_name                common_name
##  [1,] 15.67988  "Lilium candidum"         "Madonna lily"
##  [2,] 3.226063  "Lilium longiflorum"      "Easter lily"
##  [3,] 2.911383  "Allium triquetrum"       "Triquetrous garlic"
##  [4,] 2.354943  "Robinia pseudoacacia"    "False acacia"
##  [5,] 2.086884  "Ornithogalum nutans"     "Drooping star-of-Bethlehem"
##  [6,] 1.824277  "Galega officinalis"      "Goat's-rue"
##  [7,] 1.781997  "Dendrobium nobile"       "Dendrobium Stem"
##  [8,] 1.01721   "Uvularia grandiflora"    "Bellwort"
##  [9,] 0.8832858 "Hosta plantaginea"       "August-lily"
## [10,] 0.7705645 "Polygonatum odoratum"    "Solomon's-seal"
## [11,] 0.65286   "Hyacinthus orientalis"   "Hyacinth"
## [12,] 0.5609712 "Moringa oleifera"        "Drumsticktree"
## [13,] 0.5388624 "Pancratium maritimum"    "Sea-daffodil"
```

```
## [14,] 0.5148856 "Eucharis × grandiflora" NA
## [15,] 0.500501  "Paradisea liliastrum"   "St. Bruno's Lily"
```

Details on what is returned is given in the package help. In brief the score is an indication of the confidence the classifier has in its own identification. The top species is the species the classifier is most confident is represented in the image. In our study we only considered the top identification given to each image.

Image review

In our paper we undertake expert assessment of the images and the classifications in order to critically assess biases in both the images available on Flickr and the biases in the image classifier. We do this by creating a Shiny application that allowed a trained botanist to quickly go through the images and create a dataset of information about the image and the accuracy of the classification. Below is a screen shot of the app and the code used to create it.

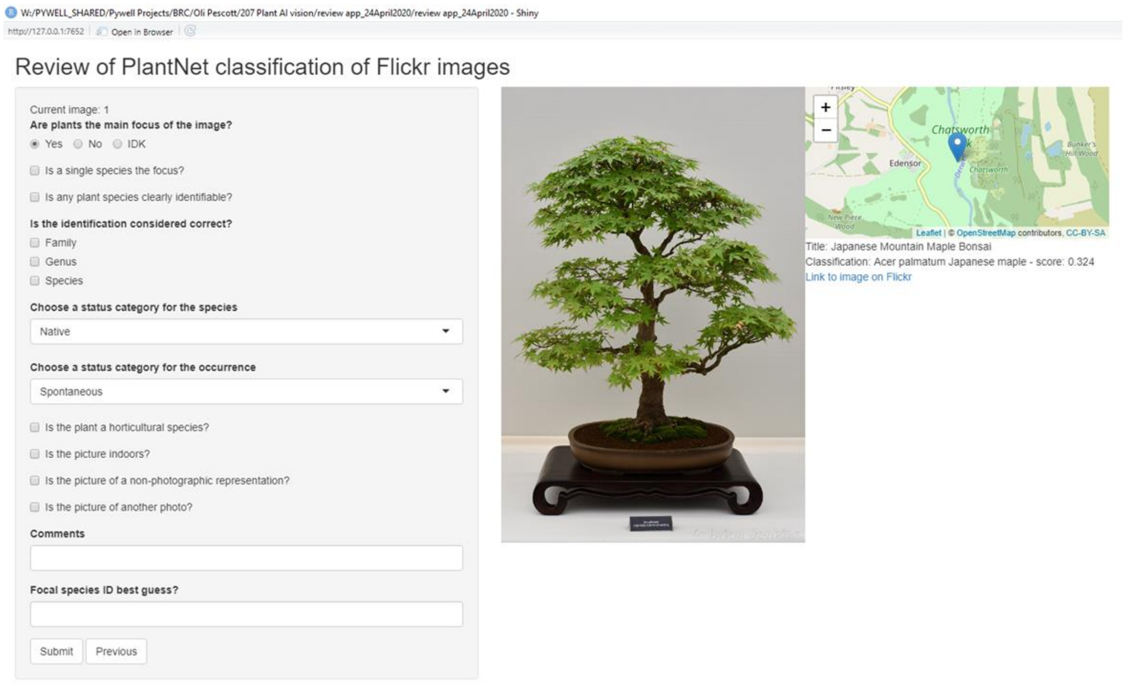

Screenshot of shiny app

```
# # Add a random number column to sample images from
# # This is added the the raw data returned from Flickr
# ids <- read.csv('data/id_results_peaks_cleaned.csv',
#                 stringsAsFactors = FALSE,
#                 header = TRUE)
# ids$rand_id <- sample(1:nrow(ids),
#                       size = nrow(ids),
#                       replace = FALSE)
# write.csv(ids,
#           file = 'data/id_results_peaks_cleaned.csv',
```

```
#            row.names = FALSE)

# Load in the data returned from Flickr
ids <- read.csv('data/id_results_peaks_cleaned.csv',
                stringsAsFactors = FALSE,
                header = TRUE)
ids <- dplyr::distinct(ids)

# Load in the table we are writing results to
# Formatted as in the excel supplementary materials
# to August et al 2020.
results <- 'results/review_results.csv'

library(shiny)
library(leaflet)

ui <- fluidPage(

    # Application title
    titlePanel("Review of PlantNet classification of Flickr images"),

    sidebarLayout(
        sidebarPanel(
            textOutput('imgID'),
            radioButtons(inputId = 'plantFocus',
                         inline = TRUE,
                         label = 'Are plants the main focus of the image?',
                         choices = c('Yes', 'No', 'IDK')),
            checkboxInput(inputId = 'plantFocSingle',
                          label = 'Is a single species the focus?',
                          value = FALSE),
            checkboxInput(inputId = 'plantIDable',
                          label = 'Is any plant species clearly
identifiable?',
                          value = FALSE),
            checkboxGroupInput(inputId = 'plantIDcorrect',
                               label = 'Is the identification considered
correct?',
                               choices = c('Family', 'Genus', 'Species')),
            selectInput(inputId = 'status_spp',
                        label = 'Choose a status category for the species',
                        choices = c('Native', 'Archaeophyte', 'Neophyte',
'NA')),
            selectInput(inputId = 'status_occ',
                        label = 'Choose a status category for the
occurrence',
                        choices = c('Spontaneous', 'Introduced', 'Unknown',
'NA')),
            checkboxInput(inputId = 'plantHort',
```

```
                            label = 'Is the plant a horticultural species?',
                            value = FALSE),
              checkboxInput(inputId = 'plantIndoor',
                            label = 'Is the picture indoors?',
                            value = FALSE),
              checkboxInput(inputId = 'plantRep',
                            label = 'Is the picture of a non-photographic
representation?',
                            value = FALSE),
              checkboxInput(inputId = 'plantMeta',
                            label = 'Is the picture of another photo?',
                            value = FALSE),
              textInput(inputId = 'comment',
                        label = 'Comments'),
              textInput(inputId = 'expertSpp',
                        label = 'Focal species ID best guess?'),
              actionButton(inputId = 'submit',
                           label = 'Submit'),
              actionButton(inputId = 'previous',
                           label = 'Previous')

        ),

        mainPanel(
           div(uiOutput("speciesImage"), style = 'float: left;'),
           leafletOutput('map', height = '200px', width = '400px'),
           textOutput('title'),
           textOutput('name'),
           htmlOutput('link')

        )
    )
)

server <- function(input, output, session) {

    imgID <- reactiveVal()
    if(file.exists(results)){
      so_far <- read.csv(results,
                         header = TRUE,
                         stringsAsFactors = FALSE)
      maxID <- min(ids$rand_id[ids$rand_id > max(as.numeric(so_far$rand_id),
                                                  na.rm = TRUE)])
      imgID(maxID)
    } else {
      imgID(min(ids$rand_id))
    }

    output$imgID <- renderText({
```

```r
      paste('Current image:', imgID())
  })

  observeEvent(input$previous, {

    imgID(max(ids$rand_id[ids$rand_id < imgID()]))

  })

  # Output image title
  output$title <- renderText({

    paste('Title:', ids$title[ids$rand_id == imgID()])

  })

  # Output the displayed image name and score
  output$name <- renderText({

    x <- ids[ids$rand_id == imgID(),
             c('latin_name',
               'common_name_english',
               'classification_score')]
    paste('Classification:', x[1,1],
          x[1,2], '- score:',
          round(x[1,3],3))
  })

  # Output link to image
  output$link <- renderUI({
    a(href = as.character(ids$image_information_link[ids$rand_id ==
imgID()]),
      'Link to image on Flickr')
  })

  # Mini-map of location
  output$map <- renderLeaflet({

    x <- ids[ids$rand_id == imgID(), ]

    if(nrow(x) > 0){

      leaflet() %>%
        addTiles() %>%
        addMarkers(lng = x$longitude,
                   lat = x$latitude)

    }
  })
```

```
observeEvent(input$submit, {

  done <- read.csv(results)

  if(!imgID() %in% done$rand_id){

    x <- ids[ids$rand_id == imgID(), ]
    x$plantFocus <- input$plantFocus
    x$plantFocSingle <- input$plantFocSingle
    x$plantIDable <- input$plantIDable
    x$plantIDcorrect <- paste(input$plantIDcorrect, collapse = ';')
    x$status_spp <- input$status_spp
    x$status_occ <- input$status_occ
    x$plantHort <- input$plantHort
    x$plantIndoor <- input$plantIndoor
    x$plantRep <- input$plantRep
    x$plantMeta <- input$plantMeta
    x$comment <- input$comment
    x$expertSpp <- input$expertSpp

    write.table(x,
                file = results,
                append = TRUE,
                col.names = FALSE,
                row.names = FALSE,
                sep = ',')

    nv <- min(ids$rand_id[ids$rand_id > imgID()])
    imgID(nv)

  } else {

    done[done$rand_id == imgID(),
         c('plantFocus', 'plantFocSingle','plantIDable',
           'plantIDcorrect','status_spp',
           'status_occ', 'plantHort','plantIndoor',
           'plantRep', 'plantMeta',
           'comment', 'expertSpp')] <- c(input$plantFocus,
                                          input$plantFocSingle,
                             input$plantIDable,
                             input$plantIDcorrect,
                             input$status_spp,
                             input$status_occ,
                             input$plantHort,
                             input$plantIndoor,
                             input$plantRep,
                             input$plantMeta,
                             input$comment,
```

```
                                input$expertSpp)

        write.table(done,
                    file = results,
                    append = FALSE,
                    col.names = TRUE,
                    row.names = FALSE,
                    sep = ',')

        nv <- min(ids$rand_id[ids$rand_id > imgID()])
        imgID(nv)
      }

      updateRadioButtons(session = session,
                         inputId = 'plantFocus',
                         inline = TRUE,
                         label = 'Are plants the main focus of the image?',
                         choices = c('Yes', 'No', 'IDK'))
      updateCheckboxInput(session = session,
                          inputId = 'plantFocSingle',
                          label = 'Is a single species the focus?',
                          value = FALSE)
      updateCheckboxGroupInput(session = session,
                               inputId = 'plantIDcorrect',
                               choices = c('Family', 'Genus', 'Species'))
      updateCheckboxInput(session = session,
                          inputId = 'plantIDable',
                          label = 'Is a plant species identifiable?',
                          value = FALSE)
      updateCheckboxInput(session = session,
                          inputId = 'plantHort',
                          label = 'Is the plant a horticultural species?',
                          value = FALSE)
      updateCheckboxInput(session = session,
                          inputId = 'plantIndoor',
                          label = 'Is the picture indoors?',
                          value = FALSE)
      updateCheckboxInput(session = session,
                          inputId = 'plantRep',
                          label = 'Is the picture of a non-photographic
representation?',
                          value = FALSE)
      updateCheckboxInput(session = session,
                          inputId = 'plantMeta',
                          label = 'Is the picture of another photo?',
                          value = FALSE)
      updateTextInput(session = session,
                      inputId = 'comment',
                      label = 'Comments',
                      value = '')
```

```r
      updateTextInput(session = session,
                      inputId = 'expertSpp',
                      label = 'Focal species ID best guess?',
                      value = '')
      updateSelectInput(session = session,
                        inputId = 'status_spp',
                        label = 'Choose a status category for the species',
                        choices = c('Native', 'Archaeophyte', 'Neophyte',
'NA'))
      updateSelectInput(session = session,
                        inputId = 'status_occ',
                        label = 'Choose a status category for the
occurrence',
                        choices = c('Spontaneous', 'Introduced', 'Unknown',
'NA'))

    })

    output$speciesImage <- renderUI({
        div(id = 'spImageDiv',
            img(src = as.character(ids$url_large_image[ids$rand_id ==
imgID()]),
                style = 'max-width: 600px; max-height: 600px')
        )
    })
}

# Run the application
shinyApp(ui = ui, server = server)
```
